# A comprehensive evaluation of 181 reported* CHST6* variants in patients with macular corneal dystrophy

**DOI:** 10.18632/aging.101807

**Published:** 2019-02-04

**Authors:** Jing Zhang, Dan Wu, Yue Li, Yidan Fan, Yiqin Dai, Jianjiang Xu

**Affiliations:** ^1^Department of Ophthalmology and Visual Science, Eye Institute, Eye and ENT Hospital, Shanghai Medical College of Fudan University, NHC Key Laboratory of Myopia (Fudan University), Shanghai Key Laboratory of Visual Impairment and Restoration, Shanghai, China

**Keywords:** *CHST6*, macular corneal dystrophy, genetic variants

## Abstract

Macular corneal dystrophy (MCD) is an autosomal recessive disease featured by bilateral progressive stromal clouding and loss of vision, consequently necessitating corneal transplantation. Variants in *CHST6* gene have been recognized as the most critical genetic components in MCD. Although many *CHST6* variants have been described until now, the detailed mechanisms underlying MCD are still far from understood. In this study, we integrated all the reported *CHST6* variants described in 408 MCD cases, and performed a comprehensive evaluation to better illustrate the causality of these variants. The results showed that majority of these variants (165 out of 181) could be classified as pathogenic or likely pathogenic. Interestingly, we also identified several disease causal variants with ethnic specificity. In addition, the results underscored the strong correlation between mutant frequency and residue conservation in the general population (Spearman’s correlation coefficient = -0.311, *P* = 1.20E-05), thus providing potential candidate targets for further genetic manipulation. The current study highlighted the demand of further functional investigations to evaluate the causality of CHST6 variants, so as to promote earlier accurate diagnosis of MCD and future development of potential targets for genetic therapy.

## INTRODUCTION

Macular corneal dystrophy (MCD; OMIM 217800) is an autosomal recessive disease featured by bilateral progressive stromal clouding and loss of vision, finally necessitating corneal transplantation [1, 2]. Cases of MCD have been recognized worldwide, while it is found to have high prevalence in India, Saudi Arabia, and Iceland [3]. The clinical symptoms usually manifest in the first decade of life, presented by a diffuse central stromal haze that progressively extends to the periphery of the cornea, which yields loss of corneal transparency and decreased vision. It is reported that MCD constitutes 10% to 75% of the corneal dystrophies that demand corneal grafting [4]. Generally, MCD could be divided into three immunophenotypes (MCD types IA, I and II) depending on the levels of keratan sulfate (KS) that detected in the serum and in the cornea. Patients with MCD type I lack KS in the serum and cornea, whilst patients with MCD type II contain detectable KS both in the serum and cornea [5]. The third type, IA, in which sulfated KS can be detected in the keratocytes instead of the serum and the cornea, has also been described [6].

Keratan sulfate plays a central role in maintaining corneal transparency. It is the major component of keratocan and lumican that are critical in collagen fibril organization. The sulfation of keratin in cornea is mediated through the corneal isoform of carbohydrate sulfotransferase 6 (CHST6), an enzyme which functions in catalyzing the transfer of a sulfate group to the GlcNAc residues of keratan [7]. The *CHST6* (OMIM 605294) gene spans approximate 23 kb of the short arm of chromosome 16 (16q23.1) and consists of 4 exons and a 1,187 bp open reading frame. The encoded protein CHST6 contains 395 amino acids with a molecular weight of 44 kDa. Like other members of the carbohydrate sulfotransferase family, it includes a short cytosolic tail at the N-terminal, a single transmembrane span, and a C-terminal domain. The sulfate donor PAPS binding site that located in the C-terminal domain determines carbohydrate specificity in vivo [8]. Deficiency in CHST6 may generate unsulfated polyactosamine chains that are less water-soluble than the fully sulfated keratan sulfate, and result in malformations in fibril organization in the cornea, which finally leads to progressive corneal opacification in MCD patients [9].

Variants in *CHST6* gene have been recognized as the most critical genetic components in MCD. To date, more than 100 frameshift, nonsense, or missense variants in *CHST6* were described in patients with MCD I/IA. In MCD II patients, large rearrangements and deletions in the upstream of* CHST6* were initially reported, followed by subsequent identification of mutations within the coding region of CHST6 [4, 9–45]. However, substantial genetic heterogeneity still exists, and there is no study systematically evaluating *CHST6* variants in MCD patients, in particular with regards to genotype-phenotype correlation and informing on the significance of specific variants.

In the current study, we conducted a comprehensive evaluation of all 181 *CHST6* variants described in MCD patients, and then classified the pathogenicity of those variants according to the American College of Medical Genetics and Genomics (ACMG) guidelines [46].

## RESULTS

### The spectrum of CHST6 variants

**Figure 1:**
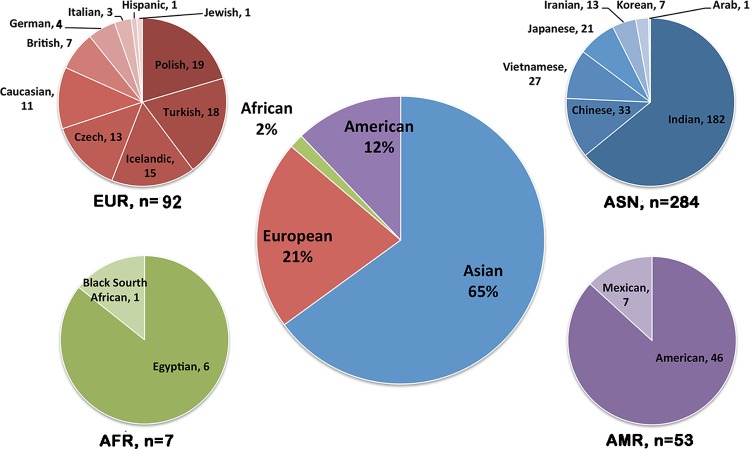
**Population distribution of *CHST6* variants found in patients with macular corneal dystrophy (MCD)**. Pie-chart showing the number of MCD patients carrying *CHST6* variants in different countries and the percentage in the general population (middle).

Totally, we retrieved information of 436 MCD cases reported in 38 articles. Most of these cases were Asian ethnicity (65%, 284 families), followed by Europeans (21%), Americans (12%) and Africans (2%, Figure 1). The age of disease onset in these reported MCD patients was highly variable, ranging from 6 to 57 years old, with the average onset age of 25.2±11.8 years old. The number of females and males were almost identical, with no obvious gender preference. Four hundred and eight MCD cases were found to harbor potential pathogenic *CHST6* variants. Among them, 270 cases had homozygous *CHST6* variants, 98 cases carried compound heterozygous variants, and 40 cases have only a single variant. A total of 181 unique *CHST6* variants were previously reported, including 128 missense, 29 frameshift, 17 nonsense, and 4 non-frameshift variants, together with 3 deletions and/or rearrangements in the upstream region of *CHST6* (Supplementary Table 1).

### Frequent CHST6 variants

**Figure 2:**
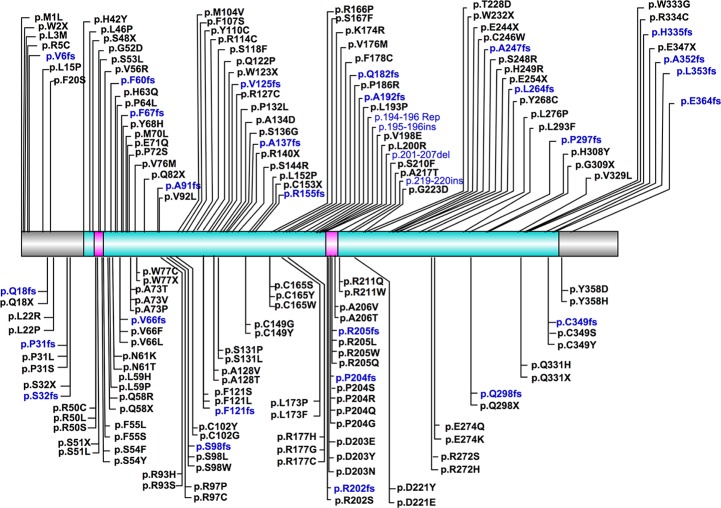
**Schematic representation of position of CHST6 variants and its protein domains**. The sulfotransferase domain (residue 42-356) was labeled in cyan, and the two PAPS binding sites (residue 49-55 and 202-210) were labeled in carmine.

The most frequently described *CHST6* variant was p.L200R, which was observed in 37 cases. This mutation was only described in Europeans and Americans, and many of the affected cases (23/37) were compound heterozygous for this mutation. The second most frequent variant was located at position 211, which has been observed in 21 cases. Three types of amino acid substitutions, including p.R211G, p.R211Q and p.R211W were identified here. Interestingly, these mutations were mainly found in Asian populations, except for a homozygous p.R211W mutation that was reported in three Turkish cases, as well as a compound heterozygous p.R211Q mutation found in a Germany patient. Of note, these frequent variants were located quite close to the sulfate donor (PAPS) binding site, implying their potential roles in affecting protein function. In general however, most of *CHST6* variants were identified in only one or a few patients, indicating the substantial genetic heterogeneity of MCD caused by *CHST6* variants. A complete list of *CHST6* variants with nucleotide and predicted amino acid changes was shown in Supplementary Table 1. The position of these variants, with regard to key domains, was illustrated in a schematic representation of the CHST6 protein (Figure 2).

**Figure 3:**
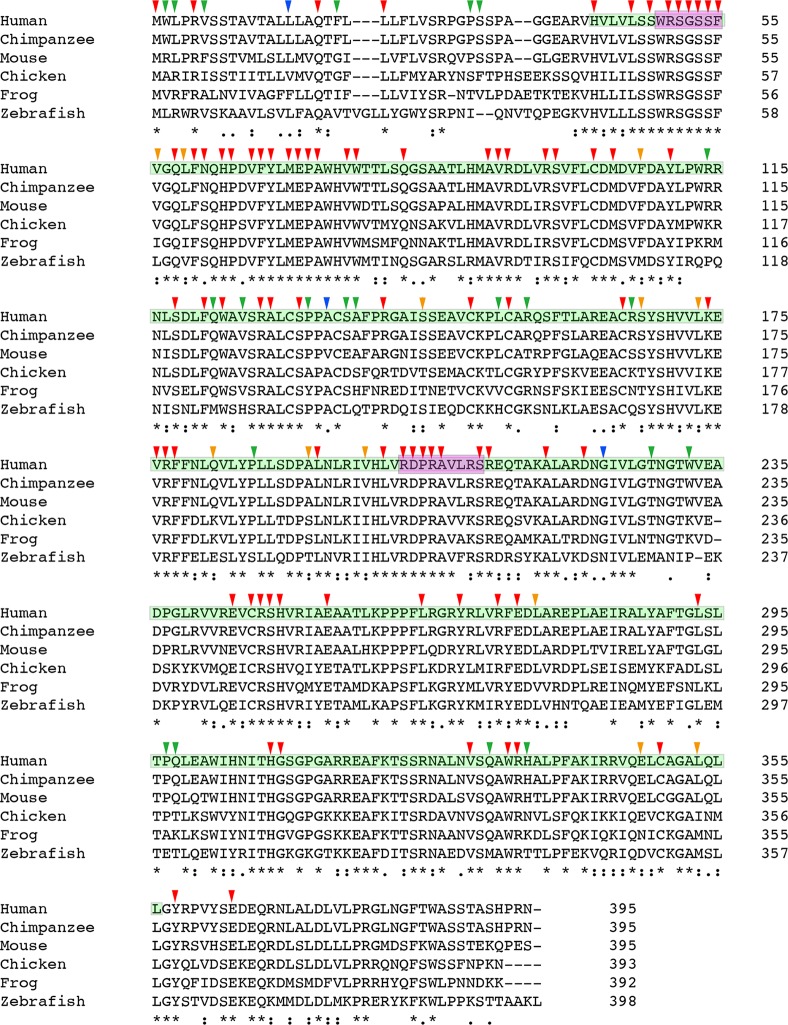
**Multiple sequence alignment result of CHST6 protein**. Protein sequences for CHST6 retrieved from NCBI for human, chimpanzee, mouse, chicken, frog and zebrafish showed amino acid conservation among different vertebrate species (for mouse, the sequence of CHST5 was used here). The sulfotransferase domain (residue 42-356) was labeled in cyan, and the two PAPS binding sites (residue 49-55 and 202-210) were labeled in carmine. Arrowheads indicated amino acid changes caused by reported human mutations. Strongly conserved positions were labeled with red or orange arrowheads, while weakly conserved ones were labeled with blue or green arrowheads (annotated by Clustal Omega).

**Figure 4:**
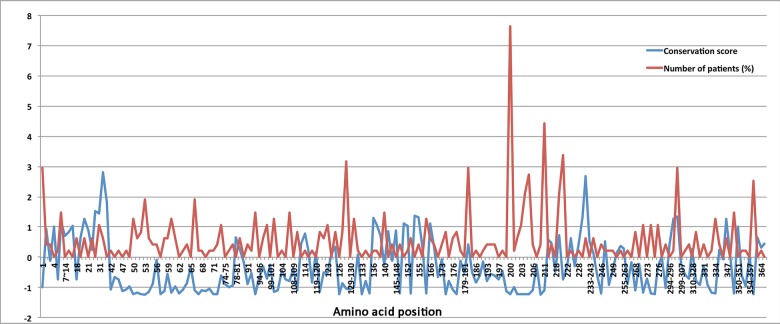
The normalized conservation scores (blue curve) for each residue in CHST6 protein and the percentage of reported MCD patients who carried mutations in the corresponding position (red curve)

Protein sequence alignment revealed that at least half of the reported *CHST6* variants that caused amino acid changes are conserved among various vertebrate species (Figure 3). Further, the normalized conservation score of each residue in the CHST6 protein was calculated using the empirical Bayesian method, as implemented in the Consurf Server (Shown in Supplementary Table 4), and compared with its mutated frequency detected in MCD patients. Both of the two measurements were transformed into z-scores before comparison. It was interesting to find that the number of patients who carried *CHST6* variants at each position was significantly correlated with the conservation score of the corresponding residue (Spearman’s correlation coefficient = -0.311, *P* = 0.000012, shown in Figure 4).

### Pathogenicity classification of all CHST6 variants

All the reported *CHST6* variants were classified based on the ACMG guidelines as described in Materials and Methods part. Finally, 62 variants were classified as pathogenic, 103 variants as likely pathogenic and 16 as being of uncertain significance.

Among the 49 protein-truncating variants, 32 of them were leading to a frameshift (including large deletions) and 17 were nonsense mutations. Majority of the truncating variants were rare, because only nine of them existed in the gnomAD database, with the frequency lower than 4.67E-05. Protein truncating variants were considered to be pathogenic if they caused a termination of the protein before residue 298, since the p.Q298X variant has already been classified as pathogenic by the ClinVar database. It thus showed that 41 of them were classified to be pathogenic.

A total of 128 missense variants in *CHST6* were identified in MCD patients, and 25 of them were classified as pathogenic by UniProt, and thereby were counted as strong evidence (PS1) of pathogenicity. Additional 31 variants occurred at the same amino acid residue with those established pathogenic variants were considered to show moderate evidence (PM5) of pathogenicity. Meanwhile, all the variants were absent from controls or at extremely low frequency in general populations, which was recognized as a moderate piece of evidence for pathogenicity (PM2). Majority of the in silico programs tested agreed on the protein-damaging prediction of these missense variants, and missense variations in *CHST6* were already recognized as a common cause of the disease, then these evidences can be counted as supporting (PP2 and PP3). Besides, the SMART prediction tool (http://smart.embl-heidelberg.de/) was applied to retrieve CHST6 protein domains and their location. It was shown that the key sulfotransferase domain ranged from residue 42 to 356, thus moderate evidence (PM2) for a variant to be pathogenic was considered if it occurred within this domain. Finally, after combining all the information in the pathogenicity evaluation, 21 missense variants were classified to be pathogenic, 100 missense variants to be likely pathogenic and 7 as being of uncertain significance. Of note, the most frequently described variant p.L200R has been classified as likely pathogenic, given the evidences of PM1, PM2, PP2, PP3 and PP5. The detailed results of ACMG classification for protein-truncating variants and missense variants were shown in Supplementary Tables 2 and 3, respectively.

## DISCUSSION

To date, many efforts have been made on the molecular diagnosis of MCD, and the implementation of next-generation sequencing (NGS) in clinical diagnosis greatly helps expand the genetic spectrums of MCD worldwide. In the current study, we performed a comprehensive evaluation on all the reported *CHST6* variants found in MCD patients, including the distribution of these variants across populations, the conservation scores among residues, the correlation between mutant frequency and residue conservation, and the potential genotype phenotype correlation. Accordingly, we further classified all the reported *CHST6* variants based on the ACMG guideline. To our knowledge, this is the first study comprehensively analyzing the genetic findings on MCD pathogenesis, and the current study may help shed light on earlier accurate diagnosis of MCD and future development of potential targets for genetic therapy.

In this study, we observed a high prevalence of MCD in Asians, in particular India, which can be attributed to the known high rate of consanguinity there [3]. We also found some causal variants with potential ethnic specificity, such as variant p.L200R and p.R211G, which have been discovered in Europeans/Americans or Asians, respectively. Importantly, we found a significant correlation between the mutant frequency and the conservation score of the corresponding residue. The top-5 most conserved residues were Ser53, Ser210, Asp203, Arg50, and Arg93, and four of them were located in the important PAPS binding site. For the most described variant p.L200R, its residue conservation score was -1.217, ranked 16. Currently, there are no animal models available that mimic human MCD, since the large number of genetic mutations in *CHST6* gene identified in MCD patients made it difficult to find a single target for genetic manipulation. However, those most conserved residues or the highly mutated residues in the general population might be potential candidates for further functional studies.

Functionally, owing to the abnormal sulfation caused by CHST6 variants, the keratin molecules cannot be metabolized and then were deposited in the cornea [47]. In addition, the collagen fibrils turned to be smaller, with a decrease in the interfibrillar spacing [48]. They together contributed to the loss of corneal transparency in MCD. It has also been demonstrated that CHST6 revealed a protective role on cell survival. For example, repression of CHST6 increased radiation-induced apoptosis of human Burkitt’s lymphoma cells [49], and more relevantly, CHST6 deficiencies were found to trigger ER stress with considerable GRP78/CHOP upregulation and cell apoptosis in MCD keratocytes [23]. All of these evidences collectively highlighted the pivotal role of CHST6 in many cellular processes, such as ECM constitution, ER stress, apoptosis and so on. Individuals with CHST6 variants might experience similar corneal dystrophy symptoms, but the underlying mechanisms for their diseases might be different at the molecular level. For example, conserved variants located within the 5’PB domain like Arg205 or Asp203 may substantially reduce the ability to combine with PAPS [50], while other conserved variants with significant changes on residue polar or physiochemical property may impact the enzymatic activity. Whether and how these crucial variants act on different cellular processes need to be clarified. Therefore, further functional investigations on some potential key variants prioritized by this study may help promote the understanding of MCD pathogenesis to a much more in-depth level.

Several limitations of this study should also be noted here. Firstly, among the 408 MCD cases that harbored pathogenic *CHST6* variants, 298 of them carried homozygous or compound heterozygous variants, fulfilling the recessive inherited model of MCD. However, there was still 40 cases carried only one single heterozygous mutation. It was quite possible that a second mutation was missed by previous sequencing methods. Thus, some state of art techniques like targeted region deep sequencing of the complete gene or whole genome sequencing can be used. In addition, for some variants that weighted to be uncertain significance in this study, additional evidences are required to push them to robustly meet the criteria of pathogenicity categories, although these data are not available at this moment.

Secondly, although we collected clinical information including age of onset, gender, and disease phenotype of MCD patients recruited in previous studies, when available. Unfortunately, approximate two thirds of patients lacked phenotypic details, and most of previous studies did not investigate the distribution and reactivity of the KS in serum and cornea in these MCD patients. Thus, we were unable to draw any conclusions with regards to the correlation between CHST6 variants and MCD immunophenotypes. The available data are still too scarce to make any correlations, implying the need for better characterization of this rare disease.

In summary, the current comprehensive evaluation contributed to the most updated *in-silico* classification of all reported CHST6 variants till now. Although the vast majority of CHST6 variants are likely to be protein damaging, systematic functional investigations are still in urgent need to demonstrate the pathogenicity of these variants.

## MATERIALS AND METHODS

### Databases and literature search

We retrieved all publications (assessed on Dec 10, 2018) from the following electronic databases: PubMed, EMBASE, and Medline, using the keywords “mutation” or “variant” combined with “CHST6” and “corneal dystrophy”. All the relevant studies, including original articles, reviews, or case reports were carefully screened. The inclusion criteria applied in this process was shown as the following: (1) sequencing studies that report CHST6 variants found in corneal dystrophy patients; (2) sufficient data for collecting variants information and disease phenotype. Reviews or obvious duplicates were removed. Variant combinations, patients’ ethnicity, and disease phenotype were collected. The number of MCD patients included in each study was recorded. Simultaneously, public databases were assessed through searches for “CHST6” in ClinVar, ClinVitae, EmvClass, and Human Gene Mutation Database (HGMD). Identified variants were compared with those from the literature search. The whole research progress was approved by the Ethics Committee of Eye and ENT Hospital of Fudan University and was performed following the declaration of Helsinki.

### Variant analysis

Each published variant was checked for accuracy and compared to the corresponding wide-type reference. When different reference sequences were used among publications, nucleotide and codon numbers were converted to ensure that their annotation matched with reference transcript NM_021615.4 for *CHST6*. The A of the ATG translation initiation codon was numbered as +1 and the initial codon as codon 1. Frequencies of the variants in control subjects were retrieved from the Genome Aggregation Database (gnomAD), which contains 123,136 exomes and 15,496 genomes from unrelated individuals worldwide (http://gnomad.broadinstitute.org).

### Classification of variant pathogenicity

We evaluated the pathogenicity of all reported *CHST6* variants according to the ACMG guidelines, to classify all variants into one of the five following categories: benign, likely benign, uncertain significance, pathogenic, or likely pathogenic. Briefly, all variants were subjected to the ANNOVAR package (http://www.openbioinformatics.org/annovar/) for variant annotation, to obtain their allele frequency in public databases, in silico prediction scores, and other evidence of pathogenicity. Then, each pathogenic criterion was evaluated to be very strong (PVS1), strong (PS1-4), moderate (PM1-6), or supporting (PP1-5), and each benign criterion was evaluated to be stand-alone (BA1), strong (BS1-4), or supporting (BP1-6). After weighting these variants based on the observed evidence of pathogenicity, the criteria were then combined to choose a classification from the five-tier system.

### Calculating the conservation scores

The conservation scores for all residues of CHST6 protein, as well as their confidence intervals were calculated by the Consurf package (http://consurf.tau.ac.il/), using the empirical Bayesian method with the default parameters. The scores were then normalized, so that the average score for all residues was zero, and the standard deviation was one. The lowest score represented the most conserved position in a protein.

## SUPPLEMENTARY MATERIAL

Supplementary Table 1

Supplementary Table 2

Supplementary Table 3

Supplementary Table 4
