# Supplementary material for: A comprehensive evaluation of 181 reported CHST6 variants in patients with macular corneal dystrophy
Source: Aging (Albany NY). 2019 Feb 4;11(3):1019–29. doi: 10.18632/aging.101807 (PMC6382428; doi:10.18632/aging.101807)
Supplement: Supplementary Table 1 [file aging-11-101807-s001.docx]

**Supplementary Table 1.** Summary of variants in *CHST6* gene.

| **Nucleotide change (NM_021615.4)** | **Amino acid change** | **Genomic coordinates (GRCh37/hg19）** | **Remarks** | **Nature of effect** | **gnomAD frequency** | **Number of families**  **/individuals** | **ACMG classification** | **Reference** |
| --- | --- | --- | --- | --- | --- | --- | --- | --- |
| c.1A>T | M1L | 16:75513726 | rs755563003 | missense | 5.09E-05 | 14 | VUS | (Klintworth et al., 2006; Gruenauer-Kloevekorn et al., 2008; Liskova et al., 2008; Dudakova et al., 2014; Nowinska et al., 2014; Yaylacioglu Tuncay et al., 2016) |
| **c.6G>A** | W2X | 16:75513721 |  | nonsense | 4.67E-05 | 2 | P | (Sultana et al., 2005; Sultana et al., 2009) |
| c.7C>A | L3M | 16:75513720 |  | missense | 4.67E-05 | 2 | VUS | (Sultana et al., 2005; Sultana et al., 2009) |
| c.13C>T | R5C | 16:75513714 |  | missense | **-** | **1** | VUS | (Dudakova et al., 2014) |
| **-** | V6fs | 16:75513712 |  | frameshift | 1.29E-05 | 7 | P | (Liu et al., 2000; Sultana et al., 2003; Warren et al., 2003; Sultana et al., 2005; Liu et al., 2006) |
| c.44T>C | L15P | 16:75513683 |  | missense | **-** | **1** | VUS | (Niel et al., 2003) |
| c.51delG | Q18fs | 16:75513676 |  | frameshift | 9.84E-06 | 1 | P | (Klintworth et al., 2006) |
| c.52C>T | Q18X | 16:75513675 |  | nonsense | **-** | **2** | P | (Sultana et al., 2003; Sultana et al., 2009) |
| c.61-62insA, 62T>G | F20fs | 16:75513666 |  | frameshift | **-** | **3** | P | (Liu et al., 2010; Wang et al., 2017) |
| c.65T>C | L22P | 16:75513666 |  | missense | **-** | **1** | LP | (El-Ashry et al., 2010) |
| c.65T>G | L22R | 16:75513662 |  | missense | **-** | **2** | P | (Warren et al., 2003; Paliwal et al., 2012) |
| c.91C>T | P31S | 16:75513636 |  | missense | **-** | **3** | LP | (El-Ashry et al., 2002; Klintworth et al., 2006) |
| c.92C>T | P31L | 16:75513635 |  | missense | **-** | **1** | LP | (Birgani et al., 2009) |
| c.97-100delTCCC | P31fs | 16:75513627-75513630 |  | frameshift | **-** | **1** | P | (Sultana et al., 2003) |
| c.95C>A | S32X | 16:75513632 |  | nonsense | **-** | **2** | P | (Liu et al., 2010; Park et al., 2015) |
| - | S32fs | 16:75513632 |  | frameshift | **-** | **1** | P | (Sultana et al., 2009) |
| c.124C>T | H42Y | 16:75513603 |  | missense | **-** | **1** | LP | (Warren et al., 2003) |
| c.137T>C | L46P | 16:75513590 |  | missense | **-** | **1** | LP | (Klintworth et al., 2006) |
| c.143C>A | S48X | 16:75513584 |  | nonsense | **-** | **1** | P | (Birgani et al., 2009) |
| c.148C>T | R50C | 16:75513579 |  | missense | **-** | **3** | P | (Akama et al., 2000; Warren et al., 2003) |
| c.148C>A | R50S | 16:75513579 |  | missense | **-** | **1** | LP | (El-Ashry et al., 2010) |
| C.149G>T | R50L | 16:75513578 | rs778668750 | missense | 7.31E-06 | 2 | LP | (Warren et al., 2003) |
| C.152C>T | S51L | 16:75513575 | rs370335460 | missense | 7.31E-06 | 2 | LP | (Ha et al., 2003b; Aldave et al., 2004) |
| C.152C>A | S51X | 16:75513575 |  | nonsense | **-** | **1** | P | (Huo et al., 2011) |
| C.155G>A | G52D | 16:75513572 |  | missense | **-** | **4** | LP | (Sultana et al., 2003; Sultana et al., 2009) |
| c.158C>T | S53L | 16:75513569 |  | missense | **-** | **9** | LP | (Sultana et al., 2003; Warren et al., 2003; Klintworth et al., 2006; Sultana et al., 2009) |
| c.161C>A | S54Y | 16:75513566 |  | missense | **-** | **1** | LP | (Paliwal et al., 2012) |
| c.161C>T | S54F | 16:75513566 |  | missense | **-** | **2** | LP | (Sultana et al., 2005; Sultana et al., 2009) |
| c.164 T>C | F55S | 16:75513563 |  | missense | **-** | **1** | LP | (Yaylacioglu Tuncay et al., 2016) |
| c.165C>A | F55L | 16:75513562 |  | missense | **-** | **1** | LP | (Birgani et al., 2009) |
| c.166G>A;c.167T>G | V56R | 16:75513560-75513561 |  | missense | **-** | **2** | LP | (Sultana et al., 2005; Sultana et al., 2009) |
| c.172C>T | Q58X | 16:75513555 | rs756036451 | nosense | 1.23E-05 | 2 | P | (Niel et al., 2003; Paliwal et al., 2012) |
| c.173A>G | Q58R | 16:75513554 |  | missense | **-** | **1** | LP | (Paliwal et al., 2012) |
| C.176T>C | L59P | 16:75513551 |  | missense | **-** | **2** | P | (Ha et al., 2003b) |
| c.176T>A | L59H | 16:75513551 |  | missense | **-** | **1** | LP | (Paliwal et al., 2012) |
| c.180delC | F60fs | 16:75513547 |  | frameshift | **-** | **6** | P | (Sultana et al., 2003; Sultana et al., 2005; Sultana et al., 2009) |
| C.182A>C | N61T | 16:75513545 |  | missense | **-** | **1** | P | (Niel et al., 2003) |
| C.183C>A | N61K | 16:75513544 |  | missense | **-** | **2** | LP | (Liu et al., 2010) |
| c.189C>G | H63Q | 16:75513538 | rs150479531 | missense | 2.05E-05 | 1 | LP | (Klintworth et al., 2006) |
| c.191C>T | P64L | 16:75513536 |  | missense | 8.20E-06 | 2 | LP | (Nowinska et al., 2014; Rubinstein et al., 2016) |
| C.196G>C | V66L | 16:75513531 |  | missense | **-** | **1** | P | (Ha et al., 2003b) |
| C.196G>T | V66F | 16:75513531 | rs72547547 | missense | 8.20E-06 | 1 | LP | (Gulias-Canizo et al., 2006) |
| C.198delC | V66fs | 16:75513529 |  | frameshift | **-** | **7** | P | (Sultana et al., 2003; Warren et al., 2003) |
| - | F67Sfs | 16:75513526 |  | frameshift | **-** | **1** | P | (Sultana et al., 2009) |
| c.202T>C | Y68H | 16:75513525 | rs775742450 | missense | 8.20E-06 | 1 | LP | (Niel et al., 2003) |
| c.208A>T | M70L | 16:75513519 |  | missense | **-** | **1** | LP | (Niel et al., 2003) |
| c.211G>C | E71Q | 16:75513516 |  | missense | **-** | **1** | LP | (Carstens et al., 2016) |
| c.214C>T | P72S | 16:75513513 | rs377617168 | missense | 4.10E-06 | 2 | LP | (El-Ashry et al., 2002; Aldave et al., 2004) |
| c.217G>C | A73P | 16:75513510 |  | missense | **-** | **1** | LP | (Klintworth et al., 2006) |
| c.217G>A | A73T | 16:75513510 |  | missense | **-** | **2** | LP | (Sultana et al., 2005; Sultana et al., 2009) |
| c.218C>T | A73V | 16:75513509 | rs776750339 | missense | 4.10E-06 | 2 | LP | (Liu et al., 2010) |
| c.226G>A | V76M | 16:75513501 |  | missense | **-** | **1** | LP | (Ha et al., 2003b) |
| c.231G>A | W77X | 16:75513496 |  | missense | 1.23E-05 | 1 | P | (Klintworth et al., 2006) |
| c.231G>C | W77C | 16:75513496 |  | missense | 3.23E-05 | 1 | LP | (Aldave et al., 2004) |
| c.244C>T | Q82X | 16:75513483 |  | nosense | **-** | **3** | P | (Ha et al., 2003a; Ha et al., 2003b; Niel et al., 2003) |
| c.271_273delGCTinsA | A91fs | 16:75513454-75513456 | rs776082747 | frameshift | 7.26E-06 | 2 | P | (Niel et al., 2003; Klintworth et al., 2006) |
| c.274G>C | V92L | 16:75513453 |  | missense | 4.09E-06 | 1 | LP | (Klintworth et al., 2006) |
| c.277C>A | R93S | 16:75513450 |  | missense | **-** | **3** | LP | (Aldave et al., 2004; Klintworth et al., 2006) |
| c.278G>A | R93H | 16:75513449 |  | missense | **-** | **4** | P | (Warren et al., 2003; Paliwal et al., 2012) |
| c.289C>T | R97C | 16:75513438 |  | missense | 4.09E-06 | 1 | LP | (Dudakova et al., 2014) |
| c.290G>C | R97P | 16:75513437 |  | missense | **-** | **2** | P | (Warren et al., 2003; Paliwal et al., 2012) |
| c.290-291 ins G | S98fs | 16:75513436-75513437 |  | frameshift | **-** | **1** | P | (Wang et al., 2017) |
| c.293C>G;c.294C>G | S98W | 16:75513433-75513434 |  | missense | **-** | **2** | LP | (Sultana et al., 2003; Sultana et al., 2009) |
| c.293C>T;c.294C>G | S98L | 16:75513433-75513434 |  | missense | **-** | **2** | LP | (Sultana et al., 2005; Sultana et al., 2009) |
| c.304T>G | C102G | 16:75513423 | rs121917822 | missense | 1.45E-05 | 4 | LP | (Niel et al., 2003; Abbruzzese et al., 2004; Aldave et al., 2004) |
| c.305G>A | C102Y | 16:75513422 |  | missense | **-** | **2** | P | (Warren et al., 2003) |
| c.310A>G | M104V | 16:75513417 |  | missense | **-** | **1** | LP | (Aldave et al., 2004) |
| c.320T>C | F107S | 16:75513407 | rs72547545 | missense | 2.86E-05 | 7 | LP | (El-Ashry et al., 2002; Sultana et al., 2003; Sultana et al., 2009) |
| c.329A>G | Y110C | 16:75513398 | rs72547544 | missense | 4.09E-06 | 4 | LP | (Aldave et al., 2004; Gonzalez-Rodriguez et al., 2014; Nowinska et al., 2014) |
| c.340C>T | R114C | 16:75513387 | rs146440274 | missense | 3.69E-05 | 1 | VUS | (Klintworth et al., 2006) |
| c.353C>T | S118F | 16:75513374 |  | missense | **-** | **1** | LP | (Park et al., 2015) |
| c.362T>C | F121S | 16:75513365 |  | missense | **-** | **1** | LP | (Sultana et al., 2009) |
| c.363_364insC | F121fs | 16:75513363-75513364 |  | frameshift | **-** | **1** | P | (Niel et al., 2003) |
| c.363C>G | F121L | 16:75513364 |  | missense | 8.19E-06 | 2 | P | (Sultana et al., 2003; Klintworth et al., 2006) |
| c.365A>C | Q122P | 16:75513362 | rs758105699 | missense | 4.10E-06 | 3 | LP | (Aldave et al., 2004; Klintworth et al., 2006) |
| c.369G>A | W123X | 16:75513358 |  | nonsense | **-** | **5** | P | (Sultana et al., 2003; Sultana et al., 2005; Sultana et al., 2009; Paliwal et al., 2012) |
| c.375_376ins(GGCCGTG) | V125fs | 16:75513351-75513352 |  | frameshift | **-** | **4** | P | (Ha et al., 2003a; Ha et al., 2003b) |
| c.379C>T | R127C | 16:75513348 |  | missense | **-** | **4** | LP | (Warren et al., 2003; Birgani et al., 2009) |
| c.382G>A | A128T | 16:75513345 |  | missense | **-** | **1** | LP | (Wang et al., 2017) |
| c.383C>T | A128V | 16:75513344 |  | missense | **-** | **14** | P | (Liu et al., 2006) |
| c.391T>C | S131P | 16:75513336 |  | missense | **-** | **3** | P | (Niel et al., 2003; Sultana et al., 2005; Sultana et al., 2009) |
| c.392C>T | S131L | 16:75513335 | rs375059043 | missense | 4.01E-05 | 3 | LP | (Klintworth et al., 2006) |
| c.395C>T | P132L | 16:75513332 |  | missense | **-** | **1** | LP | (Birgani et al., 2009) |
| c.401C>A | A134D | 16:75513326 |  | missense | 4.10E-06 | 1 | LP | (Liu et al., 2010) |
| c.406A>G | S136G | 16:75513321 |  | missense | **-** | **1** | VUS | (Birgani et al., 2009) |
| c.414insTT | A137fs | 16:75513313 |  | frameshift | **-** | **1** | P | (Akama et al., 2000) |
| c.418C>T | R140X | 16:75513309 | rs757912368 | nonsense | 8.21E-06 | 7 | P | (El-Ashry et al., 2005; Liu et al., 2005; Liu et al., 2010; Wang et al., 2017) |
| c.430A>C | S144R | 16:75513297 |  | missense | **-** | **2** | LP | (Liu et al., 2010; Wang et al., 2017) |
| c.445T>G | C149G | 16:75513281 |  | missense | **-** | **1** | LP | (Liu et al., 2010) |
| c.446G>A | C149Y | 16:75513281 |  | missense | **-** | **1** | LP | (Birgani et al., 2009) |
| - | C149fs | 16:75513280 |  | frameshift | 4.13E-06 | 1 | P | (Gruenauer-Kloevekorn et al., 2008) |
| c.455T>C | L152P | 16:75513272 |  | missense | **-** | **1** | LP | (Niel et al., 2003) |
| c.459C>A | C153X | 16:75513268 |  | nonsense | **-** | **3** | P | (Sultana et al., 2003; Sultana et al., 2005; Sultana et al., 2009) |
| c.463-464 del CG | R155Afs | 16:75513262 | rs141905571 | frameshift | 3.23E-05 | 2 | P | (Yaylacioglu Tuncay et al., 2016; Wang et al., 2017) |
| c.494G>A | C165Y | 16:75513233 |  | missense | **-** | **2** | LP | (Liskova et al., 2008; Birgani et al., 2009) |
| c.495C>G | C165W | 16:75513232 |  | missense | **-** | **2** | LP | (Sultana et al., 2005; Sultana et al., 2009) |
| c.494G>C;c.495C>T | C165S | 16:75513232-75513233 |  | missense | **-** | **2** | LP | (Sultana et al., 2005; Sultana et al., 2009) |
| c.497G>C | R166P | 16:75513230 |  | missense | **-** | **3** | LP | (Liu et al., 2000; Niel et al., 2003) |
| c.500C>T | S167F | 16:75513227 | rs756399261 | missense | 8.37E-05 | 2 | LP | (Sultana et al., 2005; Sultana et al., 2009) |
| c.517C>T | L173F | 16:75513210 | rs764287174 | missense | 4.09E-06 | 1 | LP | (Gruenauer-Kloevekorn et al., 2008) |
| c.518T>C | L173P | 16:75513209 | rs763075517 | missense | 1.23E-05 | 1 | LP | (Yellore et al., 2007) |
| c.521A>G | K174R | 16:75513206 |  | missense | **-** | **4** | LP | (Akama et al., 2000; Park et al., 2015) |
| c.526G>A | V176M | 16:75513201 |  | missense | **-** | **3** | LP | (Yaylacioglu Tuncay et al., 2016) |
| c.529C>T | R177C | 16:75513198 | rs760489234 | missense | 2.86E-05 | 2 | LP | (Klintworth et al., 2006) |
| c.529C>G | R177G | 16:75513198 |  | missense | **-** | **1** | LP | (Patel et al., 2011) |
| c.530G>A | R177H | 16:75513197 |  | missense | **-** | **1** | P | (Iida-Hasegawa et al., 2003) |
| c.533T>G | F178C | 16:75513194 |  | missense | **-** | **1** | LP | (Sultana et al., 2005) |
| c.545delA | Q182fs | 16:75513182 |  | frameshift | **-** | **14** | P | (Warren et al., 2003; Sultana et al., 2005; Sultana et al., 2009) |
| c.557C>G | P186R | 16:75513170 | rs376162109 | missense | 2.55E-05 | 2 | LP | (Patel et al., 2011; Park et al., 2015) |
| c.573_574insC | A192fs | 16:75513153 | rs570082634 | frameshift | 7.30E-06 | 1 | P | (Klintworth et al., 2006) |
| c.578T>C | L193P | 16:75513149 |  | missense | **-** | **2** | LP | (Sultana et al., 2005; Sultana et al., 2009) |
| c.581ACCTAC>GGT | 194 Asn Leu Arg 196; 194 Arg Cys 195 | 16:75513146 |  | replacement mutation | **-** | **2** | VUS | (Warren et al., 2003) |
| c.587insACG | R195-196ins | 16:75513140 |  | Insertation | **-** | **2** | VUS | (Sultana et al., 2003; Sultana et al., 2009) |
| c.593T>A | V198E | 16:75513134 |  | missense | **-** | **1** | LP | (Abbruzzese et al., 2004) |
| c.599T>G | L200R | 16:75513128 | rs28937879 | missense | 0.0002205 | 37 | LP | (El-Ashry et al., 2002; Niel et al., 2003; Abbruzzese et al., 2004; Aldave et al., 2004; El-Ashry et al., 2005; Klintworth et al., 2006; Gruenauer-Kloevekorn et al., 2008; Liskova et al., 2008; Dudakova et al., 2014; Nowinska et al., 2014) |
| c.601_621delGTGCGCGACCCGCGGGCCGTG | deletion from aa201-207 | 16:75513106-75513126 |  | deletion | **-** | **1** | VUS | (Gulias-Canizo et al., 2006) |
| c.604C>A | R202S | 16:75513123 |  | missense | **-** | **2** | P | (Sultana et al., 2003; Sultana et al., 2005) |
| c.606_607delGC | R202fs | 16:75513120-75513121 |  | frameshift | **-** | **1** | P | (El-Ashry et al., 2010) |
| c.607G>A | D203N | 16:75513120 |  | missense | **-** | **2** | LP | (Klintworth et al., 2006) |
| c.607G>T | D203Y | 16:75513120 |  | missense | **-** | **1** | LP | (Birgani et al., 2009) |
| c.609C>A | D203E | 16:75513118 |  | missense | **-** | **2** | P | (Akama et al., 2000) |
| c.610C>T | P204S | 16:75513117 |  | missense | **-** | **2** | LP | (Yaylacioglu Tuncay et al., 2016) |
| - | P204G | 16:75513116 |  | missense |  | 1 | LP | (Niel et al., 2003) |
| c.611C>A | P204Q | 16:75513116 | rs759870075 | missense | 3.23E-05 | 3 | P | (Iida-Hasegawa et al., 2003; Sultana et al., 2003; Sultana et al., 2009) |
| c.611C>G | P204R | 16:75513116 | rs759870075 | missense | 3.23E-05 | 2 | LP | (Sultana et al., 2005; Sultana et al., 2009) |
| c.612_614delGCG insAT | P204fs | 16:75513113-75513115 |  | frameshift |  | 2 | P | (Sultana et al., 2003; Warren et al., 2003) |
| c.613C>T | R205W | 16:75513114 | rs750219546 | missense | 8.38E-06 | 8 | LP | (Liu et al., 2010; Park et al., 2015) |
| c.614G>A | R205Q | 16:75513113 | rs377706989 | missense | 1.26E-05 | 3 | P | (Warren et al., 2003; Liskova et al., 2008) |
| c.614G>T | R205L | 16:75513113 |  | missense | **-** | **1** | LP | (Iida-Hasegawa et al., 2003) |
| - | R205fs | 16:75513113 |  | frameshift | **-** | **1** | P | (Sultana et al., 2009) |
| c.616G>A | A206T | 16:75513111 | rs374493344 | missense | 8.39E-06 | 1 | LP | (Warren et al., 2003) |
| c.617C>T | A206V | 16:75513110 |  | missense | **-** | **1** | LP | (El-Ashry et al., 2002) |
| c.629C>T | S210F | 16:75513098 | rs745571211 | missense | 1.68E-05 | 2 | LP | (Sultana et al., 2003; Sultana et al., 2009) |
| c.631C>G | R211G | 16:75513096 |  | missense | **-** | **1** | LP | (Liu et al., 2010) |
| c.631C>T | R211W | 16:75513096 |  | missense | **-** | **8** | P | (Akama et al., 2000; Iida-Hasegawa et al., 2003; Yaylacioglu Tuncay et al., 2016) |
| c.632G>A | R211Q | 16:75513095 | rs771397083 | missense | 4.21E-06 | 12 | LP | (Ha et al., 2003a; Ha et al., 2003b; Gruenauer-Kloevekorn et al., 2008; Patel et al., 2011) |
| c.649G>A | A217T | 16:75513078 | rs752785520 | missense | 4.21E-06 | 2 | LP | (Iida-Hasegawa et al., 2003; Kobayashi et al., 2007) |
| c.656insCTG | W219-220ins | 16:75513071 |  | Insertion | **-** | **10** | VUS | (Sultana et al., 2003; Sultana et al., 2009) |
| c.661G>T | D221Y | 16:75513066 |  | missense | **-** | **6** | LP | (Sultana et al., 2003; Sultana et al., 2005; Sultana et al., 2009) |
| c.663C>G | D221E | 16:75513064 |  | missense | **-** | **10** | P | (Sultana et al., 2003; Sultana et al., 2005; Sultana et al., 2009) |
| c.668G>A | G223D | 16:75513059 |  | missense | **-** | **1** | LP | (Liu et al., 2005) |
| c.682A>G; 683C>A | T228D | 16:75513044-75513045 |  | missense | **-** | **1** | VUS | (Klintworth et al., 2006) |
| c.696G>A | W232X | 16:75513031 |  | nonsense | **-** | **3** | P | (Ha et al., 2003b; Huo et al., 2011) |
| c.730G>T | E244X | 16:75512997 |  | nonsense | **-** | **3** | P | (Liu et al., 2010; Wang et al., 2017) |
| c.738C>G | C246W | 16:75512989 |  | missense | 3.24E-05 | 2 | LP | (Klintworth et al., 2006; Yaylacioglu Tuncay et al., 2016) |
| c.740delG | A247fs | 16:75512987 |  | frameshift | **-** | **1** | P | (Klintworth et al., 2006) |
| c.744C>G | S248R | 16:75512983 | rs760287510 | missense | 4.29E-06 | 1 | LP | (Klintworth et al., 2006) |
| c.746A>C | H249P | 16:75512981 | rs72547540 | missense | 3.24E-05 | 1 | P | (Warren et al., 2003) |
| c.746A>G | H249R | 16:75512981 |  | missense | **-** | **1** | LP | (Birgani et al., 2009) |
| c.760G>T | E254X | 16:75512967 |  | nonsense | **-** | **1** | P | (Liu et al., 2010) |
| c.786delC | L264fs | 16:75512941 |  | frameshift | **-** | **1** | P | (Park et al., 2015) |
| c.803A>G | Y268C | 16:75512924 | rs72547539 | missense | 6.64E-05 | 4 | LP | (Ha et al., 2003b; Liu et al., 2010) |
| c.814C>A | R272S | 16:75512913 | rs769833756 | missense | 4.15E-06 | 4 | LP | (Sultana et al., 2005; Sultana et al., 2009; Liu et al., 2010) |
| c.815G>A | R272H | 16:75512912 | rs745966069 | missense | 4.15E-06 | 1 | LP | (Klintworth et al., 2006) |
| c.820G>A | E274K | 16:75512907 | rs72547538 | missense | 4.14E-06 | 4 | P | (Akama et al., 2000; Warren et al., 2003; El-Ashry et al., 2010; Park et al., 2015) |
| c.820G>C | E274Q | 16:75512907 |  | missense | **-** | **1** | LP | (Paliwal et al., 2012) |
| c.827T>C | L276P | 16:75512900 | rs121917824 | missense | 5.49E-05 | 5 | LP | (Aldave et al., 2004; El-Ashry et al., 2005; Sultana et al., 2005; Sultana et al., 2009) |
| c.877C>T | L293F | 16:75512850 |  | missense | **-** | **2** | LP | (Paliwal et al., 2012) |
| c.891-892insC | P297fs | 16:75512835-75512836 |  | frameshift | **-** | **1** | P | (Birgani et al., 2009) |
| c.892C>T | Q298X | 16:75512835 |  | nonsense | **-** | **9** | P | (Dang et al., 2009; Liu et al., 2010; Wang et al., 2017) |
| c.894_895 insG | Q298fs | 16:75512832-16:75512833 |  | frameshift | **-** | **5** | P | (Yaylacioglu Tuncay et al., 2016) |
| c.922C>T | H308Y | 16:75512805 |  | missense | **-** | **1** | LP | (Park et al., 2015) |
| c.925G>T | G309X | 16:75512802 |  | nonsense | **-** | **2** | LP | (Sultana et al., 2003; Sultana et al., 2009) |
| c.985G>C | V329L | 16:75512742 |  | missense | **-** | **4** | LP | (Liu et al., 2006) |
| c.991C>T | Q331X | 16:75512736 |  | nonsense | **-** | **1** | LP | (Aldave et al., 2004) |
| c.993G>T | Q331H | 16:75512734 | rs140699573 | missense | 0.003734 | 1 | LP | (Liu et al., 2005) |
| c.997T>G | W333G | 16:75512730 |  | missense | **-** | **1** | LP | (Liu et al., 2010) |
| c.1000C>T | R334C | 16:75512727 |  | missense | 4.07E-06 | 6 | LP | (Sultana et al., 2005; Gulias-Canizo et al., 2006; Klintworth et al., 2006; Sultana et al., 2009) |
| - | H335fs | 16:75512724 |  | frameshift | **-** | **2** | VUS | (Sultana et al., 2009) |
| c.1039G>T | E347X | 16:75512688 |  | nonsense | **-** | **3** | LP | (Sultana et al., 2003; Sultana et al., 2009) |
| c.1043_1044delTG | C349fs | 16:75512683-75512684 |  | frameshift | **-** | **1** | VUS | (Gruenauer-Kloevekorn et al., 2008) |
| c.1045T>A | C349S | 16:75512682 |  | missense | **-** | **2** | LP | (Liu et al., 2010) |
| c.1046G>A | C349Y | 16:75512681 | rs747897373 | missense | 1.22E-05 | 4 | LP | (Klintworth et al., 2006; Dudakova et al., 2014) |
| c.1044_1051dupGTGCGCTG | L353fs | 16:75512676-75512683 |  | frameshift | **-** | **1** | VUS | (Aldave et al., 2004) |
| c.1056_1078delGCTGCAGCTGCTGGGCTACCGGC | A352fs | 16:75512649-75512671 |  | frameshift | **-** | **1** | VUS | (Warren et al., 2003) |
| c.1072T>C | Y358H | 16:75512655 |  | missense | **-** | **11** | LP | (Dang et al., 2009; Liu et al., 2010; Park et al., 2015; Wang et al., 2017) |
| c.1072T>G | Y358D | 16:75512655 |  | missense | **-** | **1** | P | (El-Ashry et al., 2005) |
| c.1089_1090delTG | E364fs | 16:75512637-75512638 |  | frameshift | **-** | **1** | VUS | (Rubinstein et al., 2016) |
| Deletion Including ORF |  |  |  | entire |  | 7 | P | (Akama et al., 2000; Warren et al., 2003; Klintworth et al., 2006; Young et al., 2007; El-Ashry et al., 2010) |
| Replacement of 5'region |  |  |  | entire |  | 3 | P | (Akama et al., 2000) |
| Deletion of 5'region |  |  |  | entire |  | 3 | P | (Abbruzzese et al., 2004) |

Abbruzzese, C., Kuhn, U., Molina, F., Rama, P. and De Luca, M., 2004. Novel mutations in the CHST6 gene causing macular corneal dystrophy. Clin Genet 65, 120-5.

Akama, T.O., Nishida, K., Nakayama, J., Watanabe, H., Ozaki, K., Nakamura, T., Dota, A., Kawasaki, S., Inoue, Y., Maeda, N., Yamamoto, S., Fujiwara, T., Thonar, E.J., Shimomura, Y., Kinoshita, S., Tanigami, A. and Fukuda, M.N., 2000. Macular corneal dystrophy type I and type II are caused by distinct mutations in a new sulphotransferase gene. Nat Genet 26, 237-41.

Aldave, A.J., Yellore, V.S., Thonar, E.J., Udar, N., Warren, J.F., Yoon, M.K., Cohen, E.J., Rapuano, C.J., Laibson, P.R., Margolis, T.P. and Small, K., 2004. Novel mutations in the carbohydrate sulfotransferase gene (CHST6) in American patients with macular corneal dystrophy. Am J Ophthalmol 137, 465-73.

Birgani, S.A., Salehi, Z., Houshmand, M., Mohamadi, M.J., Promehr, L.A. and Mozafarzadeh, Z., 2009. Novel mutations of CHST6 in Iranian patients with macular corneal dystrophy. Mol Vis 15, 373-7.

Carstens, N., Williams, S., Goolam, S., Carmichael, T., Cheung, M.S., Buchmann-Moller, S., Sultan, M., Staedtler, F., Zou, C., Swart, P., Rice, D.S., Lacoste, A., Paes, K. and Ramsay, M., 2016. Novel mutation in the CHST6 gene causes macular corneal dystrophy in a black South African family. BMC Med Genet 17, 47.

Dang, X., Zhu, Q., Wang, L., Su, H., Lin, H., Zhou, N., Liang, T., Wang, Z., Huang, S., Ren, Q. and Qi, Y., 2009. Macular corneal dystrophy in a Chinese family related with novel mutations of CHST6. Mol Vis 15, 700-5.

Dudakova, L., Palos, M., Svobodova, M., Bydzovsky, J., Huna, L., Jirsova, K., Hardcastle, A.J., Tuft, S.J. and Liskova, P., 2014. Macular corneal dystrophy and associated corneal thinning. Eye (Lond) 28, 1201-5.

El-Ashry, M.F., Abd El-Aziz, M.M., Shalaby, O. and Bhattacharya, S.S., 2010. Molecular genetic study of Egyptian patients with macular corneal dystrophy. Br J Ophthalmol 94, 250-5.

El-Ashry, M.F., Abd El-Aziz, M.M., Shalaby, O., Wilkins, S., Poopalasundaram, S., Cheetham, M., Tuft, S.J., Hardcastle, A.J., Bhattacharya, S.S. and Ebenezer, N.D., 2005. Novel CHST6 nonsense and missense mutations responsible for macular corneal dystrophy. Am J Ophthalmol 139, 192-3.

El-Ashry, M.F., Abd El-Aziz, M.M., Wilkins, S., Cheetham, M.E., Wilkie, S.E., Hardcastle, A.J., Halford, S., Bayoumi, A.Y., Ficker, L.A., Tuft, S., Bhattacharya, S.S. and Ebenezer, N.D., 2002. Identification of novel mutations in the carbohydrate sulfotransferase gene (CHST6) causing macular corneal dystrophy. Invest Ophthalmol Vis Sci 43, 377-82.

Gonzalez-Rodriguez, J., Ramirez-Miranda, A., Hernandez-Da Mota, S.E. and Zenteno, J.C., 2014. TGFBI, CHST6, and GSN gene analysis in Mexican patients with stromal corneal dystrophies. Graefes Arch Clin Exp Ophthalmol 252, 1267-72.

Gruenauer-Kloevekorn, C., Braeutigam, S., Heinritz, W., Froster, U.G. and Duncker, G.I., 2008. Macular corneal dystrophy: mutational spectrum in German patients, novel mutations and therapeutic options. Graefes Arch Clin Exp Ophthalmol 246, 1441-7.

Gulias-Canizo, R., Castaneda-Diez, R., Gomez-Leal, A., Klintworth, G.K. and Rodriguez-Reyes, A.A., 2006. [Corneal macular dystrophy: clinical, histopathologic and ultrastructural features]. Arch Soc Esp Oftalmol 81, 315-20.

Ha, N.T., Chau, H.M., Cung le, X., Thanh, T.K., Fujiki, K., Murakami, A., Hiratsuka, Y., Hasegawa, N. and Kanai, A., 2003a. Identification of novel mutations of the CHST6 gene in Vietnamese families affected with macular corneal dystrophy in two generations. Cornea 22, 508-11.

Ha, N.T., Chau, H.M., Cung le, X., Thanh, T.K., Fujiki, K., Murakami, A., Hiratsuka, Y. and Kanai, A., 2003b. Mutation analysis of the carbohydrate sulfotransferase gene in Vietnamese with macular corneal dystrophy. Invest Ophthalmol Vis Sci 44, 3310-6.

Huo, Y.N., Yao, Y.F. and Yu, P., 2011. Pathogenic mutations of TGFBI and CHST6 genes in Chinese patients with Avellino, lattice, and macular corneal dystrophies. J Zhejiang Univ Sci B 12, 687-93.

Iida-Hasegawa, N., Furuhata, A., Hayatsu, H., Murakami, A., Fujiki, K., Nakayasu, K. and Kanai, A., 2003. Mutations in the CHST6 gene in patients with macular corneal dystrophy: immunohistochemical evidence of heterogeneity. Invest Ophthalmol Vis Sci 44, 3272-7.

Klintworth, G.K., Smith, C.F. and Bowling, B.L., 2006. CHST6 mutations in North American subjects with macular corneal dystrophy: a comprehensive molecular genetic review. Mol Vis 12, 159-76.

Kobayashi, A., Fujiki, K., Fujimaki, T., Murakami, A. and Sugiyama, K., 2007. In vivo laser confocal microscopic findings of corneal stromal dystrophies. Arch Ophthalmol 125, 1168-73.

Liskova, P., Veraitch, B., Jirsova, K., Filipec, M., Neuwirth, A., Ebenezer, N.D., Hysi, P.G., Hardcastle, A.J., Tuft, S.J. and Bhattacharya, S.S., 2008. Sequencing of the CHST6 gene in Czech macular corneal dystrophy patients supports the evidence of a founder mutation. Br J Ophthalmol 92, 265-7.

Liu, N.P., Bao, W., Smith, C.F., Vance, J.M. and Klintworth, G.K., 2005. Different mutations in carbohydrate sulfotransferase 6 (CHST6) gene cause macular corneal dystrophy types I and II in a single sibship. Am J Ophthalmol 139, 1118-20.

Liu, N.P., Dew-Knight, S., Rayner, M., Jonasson, F., Akama, T.O., Fukuda, M.N., Bao, W., Gilbert, J.R., Vance, J.M. and Klintworth, G.K., 2000. Mutations in corneal carbohydrate sulfotransferase 6 gene (CHST6) cause macular corneal dystrophy in Iceland. Mol Vis 6, 261-4.

Liu, N.P., Smith, C.F., Bowling, B.L., Jonasson, F. and Klintworth, G.K., 2006. Macular corneal dystrophy types I and II are caused by distinct mutations in the CHST6 gene in Iceland. Mol Vis 12, 1148-52.

Liu, Z., Tian, X., Iida, N., Fujiki, K., Xie, P., Wang, W., Ma, Z., Kanai, A. and Murakami, A., 2010. Mutation analysis of CHST6 gene in Chinese patients with macular corneal dystrophy. Cornea 29, 883-8.

Niel, F., Ellies, P., Dighiero, P., Soria, J., Sabbagh, C., San, C., Renard, G., Delpech, M. and Valleix, S., 2003. Truncating mutations in the carbohydrate sulfotransferase 6 gene (CHST6) result in macular corneal dystrophy. Invest Ophthalmol Vis Sci 44, 2949-53.

Nowinska, A.K., Wylegala, E., Teper, S., Wroblewska-Czajka, E., Aragona, P., Roszkowska, A.M., Micali, A., Pisani, A. and Puzzolo, D., 2014. Phenotype and genotype analysis in patients with macular corneal dystrophy. Br J Ophthalmol 98, 1514-21.

Paliwal, P., Sharma, A., Tandon, R., Sharma, N., Titiyal, J.S., Sen, S. and Vajpayee, R.B., 2012. Molecular genetic analysis of macular corneal dystrophy patients from North India. Ophthalmic Res 48, 28-32.

Park, S.H., Ahn, Y.J., Chae, H., Kim, Y., Kim, M.S. and Kim, M., 2015. Molecular analysis of the CHST6 gene in Korean patients with macular corneal dystrophy: Identification of three novel mutations. Mol Vis 21, 1201-9.

Patel, D.A., Harocopos, G.J., Chang, S.H., Vora, S.C., Lubniewski, A.J. and Huang, A.J., 2011. Novel CHST6 gene mutations in 2 unrelated cases of macular corneal dystrophy. Cornea 30, 664-9.

Rubinstein, Y., Weiner, C., Einan-Lifshitz, A., Chetrit, N., Shoshany, N., Zadok, D., Avni, I. and Pras, E., 2016. Macular Corneal Dystrophy and Posterior Corneal Abnormalities. Cornea 35, 1605-1610.

Sultana, A., Klintworth, G.K., Thonar, E.J., Vemuganti, G.K. and Kannabiran, C., 2009. Immunophenotypes of macular corneal dystrophy in India and correlation with mutations in CHST6. Mol Vis 15, 319-25.

Sultana, A., Sridhar, M.S., Jagannathan, A., Balasubramanian, D., Kannabiran, C. and Klintworth, G.K., 2003. Novel mutations of the carbohydrate sulfotransferase-6 (CHST6) gene causing macular corneal dystrophy in India. Mol Vis 9, 730-4.

Sultana, A., Sridhar, M.S., Klintworth, G.K., Balasubramanian, D. and Kannabiran, C., 2005. Allelic heterogeneity of the carbohydrate sulfotransferase-6 gene in patients with macular corneal dystrophy. Clin Genet 68, 454-60.

Wang, L., Tang, X., Lv, X., Sun, E., Wu, D., Wang, C. and Liu, P., 2017. CHST6 mutation screening and endoplasmatic reticulum stress in macular corneal dystrophy. Oncotarget 8, 96301-96312.

Warren, J.F., Aldave, A.J., Srinivasan, M., Thonar, E.J., Kumar, A.B., Cevallos, V., Whitcher, J.P. and Margolis, T.P., 2003. Novel mutations in the CHST6 gene associated with macular corneal dystrophy in southern India. Arch Ophthalmol 121, 1608-12.

Yaylacioglu Tuncay, F., Kayman Kurekci, G., Guntekin Ergun, S., Pasaoglu, O.T., Akata, R.F. and Dincer, P.R., 2016. Genetic analysis of CHST6 and TGFBI in Turkish patients with corneal dystrophies: Five novel variations in CHST6. Mol Vis 22, 1267-1279.

Yellore, V.S., Sonmez, B., Chen, M.C., Rayner, S.A., Thonar, E.J. and Aldave, A.J., 2007. An unusual presentation of macular corneal dystrophy associated with uniparental isodisomy and a novel Leu173Pro mutation. Ophthalmic Genet 28, 169-74.

Young, R.D., Akama, T.O., Liskova, P., Ebenezer, N.D., Allan, B., Kerr, B., Caterson, B., Fukuda, M.N. and Quantock, A.J., 2007. Differential immunogold localisation of sulphated and unsulphated keratan sulphate proteoglycans in normal and macular dystrophy cornea using sulphation motif-specific antibodies. Histochem Cell Biol 127, 115-20.
